# Draft Genome Sequence of an Aldoxime Degrader, *Rhodococcus* sp. Strain YH3-3

**DOI:** 10.1128/genomeA.00406-16

**Published:** 2016-05-19

**Authors:** Takuya Yamaguchi, Yasuhisa Asano

**Affiliations:** a Biotechnology Research Center and Department of Biotechnology, Toyama Prefectural University, Imizu, Toyama, Japan; b Asano Active Enzyme Molecule Project, ERATO, JST, Imizu, Toyama, Japan

## Abstract

*Rhodococcus* sp. strain YH3-3 has been isolated as an (*E*)-pyridine-3-aldoxime degrader. Here, we report the draft genome sequence of this strain, with a size of 7,316,908 bp, average G+C content of 62.15%, and 7,281 predicted protein-coding sequences.

## GENOME ANNOUNCEMENT

Nitriles and nitrile-hydrolyzed products, such as amides and carboxylic acids, are extensively used in the chemical industry (1). Because the enzymatic reaction is environmentally friendly, nitrile hydratase (NHase) and amidase (Ami) are used in the industrial production of amides from nitriles and carboxylic acids from amides, respectively (2, 3). The recombinant aldoxime dehydratase (Oxd) from an aldoxime degrader, *Bacillus* sp. strain OxB-1, has been applied to the nitrile synthesis (4, 5). *Rhodococcus* sp. strain YH3-3 was isolated as an (*E*)-pyridine-3-aldoxime-degrading bacterium (6). This strain metabolizes (*E*)-pyridine-3-aldoxime to nicotinic acid via 3-pyridinecarbonitrile and nicotinamide using a combination of Oxd, NHase, and Ami (6, 7). A cobalt-type NHase has been purified and characterized from this strain (7); however, Oxd and Ami have not been identified from this strain. In this study, genome sequencing was performed to identify the enzymes in the nitrile metabolism pathway of *Rhodococcus* sp. strain YH3-3.

The draft genome of the strain YH3-3 was sequenced by a shotgun strategy using Illumina Hiseq 2000 (Illumina, Hayward, CA), which produced paired-end and mate-paired reads totaling ~2,200 Mb with approximately 300-fold coverage of the genome. The genome sequence data were processed, assembled into 97 contigs, and generated 38 scaffolds (*N*_50_ = 260,2841 bp) using SOAPdenovo version 1.05 (8). The genome sequence was annotated using the Glimmer 3.0 (9) for protein-coding sequence (CDS) prediction, RNAmmer (10) for rRNA prediction, tRNAscan-SE (11) for tRNA prediction, and BLAST (12) for a homology search.

The draft genome of strain YH3-3 was 7,316,908 bp in length with G+C content of 62.15%, containing 7,281 open reading frames, 54 tRNA genes, and 5 rRNA operons. In total, 6,893 CDSs matched known genes (94.6% of all CDSs). The draft genome contained two gene clusters responsible for nitrile metabolism. One gene cluster contained *oxd*, iron-type *nhase*, and *ami*. The other contained cobalt-type *nhase* and *ami*, but not *oxd*. The N-terminal sequence obtained from previously purified NHase (7) corresponded to that of cobalt-type NHase. Furthermore, a gene encoding nitrilase, which catalyzes the hydration of nitriles to carboxylic acids, was observed. This gene is clustered with *oxd* in *Bacillus* sp. strain OxB-1 (13), but not in the strain YH3-3. Thus, six genes encoding nitrile-metabolizing enzymes were identified from the draft genome sequence of *Rhodococcus* sp. strain YH3-3. These enzymes could be applied to biocatalytic synthesis of nitriles, amides, and carboxylic acids.

### Nucleotide sequence accession numbers. 

The data from this draft-genome shotgun project have been deposited in DDBJ/EMBL/GenBank under the accession number BCWH00000000. The version described in this paper is the first version, BCWH01000000.
